# Preparing a cost analysis for the section of medical physics–guidelines and methods

**DOI:** 10.1120/jacmp.v1i2.2648

**Published:** 2000-03-01

**Authors:** Michael D. Mills, William J. Spanos, Baby O. Jose, Beverly A. Kelly, James P. Brill

**Affiliations:** ^1^ Department of Radiation Oncology University of Louisville 529 South Jackson Street Louisville Kentucky 40202; ^2^ University of Louisville Hospital 530 South Jackson Street Louisville Kentucky 40202

**Keywords:** audit preparation, compliance program, cost report, manpower, work units

## Abstract

Radiation oncology is a highly complex medical specialty, involving many varied routine and special procedures. To assure cost‐effectiveness and maintain support for the medical physics program, managers are obligated to analyze and defend all aspects of an institutional billing and cost‐reporting program. Present standards of practice require that each patient's radiation treatments be customized to fit his/her particular condition. Since the use of personnel time and other resources is highly variable among patients, graduated levels of charges have been established to allow for more precise billing. Some radiation oncology special procedures have no specific code descriptors; so existing codes are modified or additional information attached in order to avoid payment denial. Recent publications have explored the manpower needs, salaries, and other resources required to perform radiation oncology “physics” procedures. This information is used to construct a model cost‐based resource use profile for a radiation oncology center. This profile can be used to help the financial officer prepare a cost report for the institution. Both civil and criminal penalties for Medicare fraud and abuse (intentional or unintentional) are included in the False Claims Act and other statutes. Compliance guidelines require managers to train all personnel in correct billing procedures and to review continually billing performance. © *2000 American College of Medical Physics.*

PACS number(s): 87.90+y, 87.53.–j

## INTRODUCTION

Institutions such as hospitals, skilled nursing facilities, and home health agencies are reimbursed in part on the basis of their self‐reported operating costs. A hospital's financial health is therefore highly dependent on the ability of the financial officers to identify and report all costs associated with operating and providing medical services. A radiation oncology manager can assist the financial officer by providing estimated or documented costs of equipment, supplies, operations, salaries, and workloads associated with individual codes. This report develops a mechanism to communicate this information to the hospital's financial officers. The 773XX “Physics” codes (see below) are used to illustrate this mechanism.

It is standard practice for hospitals to develop some internal mechanism to capture and record the work associated with the services provided for patients. Most financial officers are open to using published industry standards of estimating work relative value units (work RVU). Technical work is measured in units of time, unmodified by considerations of intensity of work or other factors. Work RVU values are usually expressed in units of hours; however, occasionally other units such as minutes or quarter hour units have been used. This report will use hourly units to report personnel time.

Patient billing codes for every medical service provided to a patient are listed in the *Current Procedural Terminology (CPT), Fourth Edition.* ^1^ Billing codes are listed and described in this document. This listing is reviewed and modified each year by the CPT Editorial Panel of the American Medical Association. The codes beginning with the number 773 are considered to be the radiation oncology “physics” codes. Revenue from these codes is intended to reimburse the efforts and costs of physicians, physicists, dosimetrists, and institutions that provide these services.

While there is a uniform language to describe medical services, the CPT, there is no national set of billing policies for Medicare. Each local Medicare carrier experiences some freedom to establish its own policies, as do most of the private insurance carriers. Any provider should have as a goal the establishment of accurate, precise, equitable, and consistent billing policies, and establish a track record of honesty and fairness with respect to billing. A Medicare carrier may decide to publish and distribute a copy of reimbursement policies for radiation oncology. Recently, the carrier for Colorado published a draft of proposed policies for comment and review. If possible, it is desirable to obtain a copy of the policy used by the local Medicare intermediary.

## METHODS AND ANALYSIS

Dosimetrist and medical physicist times as well as median service volume for the 773XX CPT codes were measured as part of the *Abt Study of Medical Physicist Work Values for Radiation Oncology Physics Services*,^2^ and are reported in Table I. These values are for routine procedures only; special procedures were not measured as part of the Abt Study. The dosimetrist hours were not tabulated in the published final report, however these values were measured, validated, and approved during the course of the study and are reported here. The distribution according to practice type indicated 44% of responding medical physicists practiced in private or community hospitals, 33% in medical schools or university hospitals, 10% in medical physics consulting groups, 10% in physician groups, and 3% in government hospitals. Volume numbers are illustrative for a clinic treating approximately 400 patients per year, however are not reflective of any particular clinic year. The hourly work effort for dosimetry and physics is adjusted according to volume, and salary costs for each procedure are calculated. According to the *American Association of Physicists in Medicine Professional Information Survey Report, Calendar Year 1998*,^3^ the average total income for a medical physicist in the United States was $99,000, while income at the 80th percentile was approximately 30% higher. This indicates the range of salaries an employer might expect to pay in order to provide high‐end services. Adjusting the average total income figure by 1.3 for benefits, and again by 1.1 for recruiting and other contingencies, the salary basis of the average medical physicist is $141,570.00 per year. The American Association of Medical Dosimetrists published a *Salary Survey for Calendar Year 1997*,^4^ which indicated the average salary for a dosimetrist in the United States was $52,050. The annual salary basis for a medical dosimetrist is therefore ($52,050) (1.3) (1.1) = $74,432. For this report, the salary basis for the dosimetrist is established at $35.00 per hour, and the salary basis of the physicist is $70.00 per hour. However salary and all other information used in a similar clinic specific analysis must reflect the costs actually accrued by the hospital or institution. The equipment depreciation for routine services is estimated assuming $500,000 in equipment expense, including the treatment‐planning computer, beam scanner, thermoluminescent dosimeter (TLD) reader, and other specialized equipment. These capital expenses are depreciated over 5 years in this analysis. Since only the physicist and dosimetrist use the equipment, depreciation is calculated as $500,000/(2080 hours/person−year×2 people×5 year)=$24.00 per service hour for both dosimetrist and physicist work. The sum of salary and equipment depreciation is expressed as the direct cost of performing the service. Indirect costs, including space, utilities, cleaning, parking, laundry, office supplies and etc. are calculated here as 40% of direct costs. Again, each of these values will be institution specific. The financial officer is required to use actual salary information, true equipment costs, and a measured indirect to direct cost ratio in the preparation of the cost report. Total costs can be used to defend both the cost and value of providing radiation oncology services to a community, and are exemplified in Table II.

**Table I:** Salary costs associated with providing radiation oncology physics routine services.

CPT code	Dosim: hours	Phys hours	Volume annual	Vol adj RVU dos	Vol adj RVU phy	Sal cost dosim	Sal cost phys	Total sal cost
77300	0.25	0.63	600	150.00	378.00	$5,250.00	$26,460.00	$31,710.00
77305	1	0.82	20	20.00	16.40	$700.00	$1,148.00	$1,848.00
77310	1.3	0.93	41	53.30	38.13	$1,865.50	$2,669.10	$4,534.60
77315	2	1.15	190	380.00	218.50	$13,300.00	$15,295.00	$28,595.00
77321	2	1.21	5	10.00	6.05	$350.00	$423.50	$773.50
77326	1	2.13	5	5.00	10.65	$175.00	$745.50	$920.50
77327	1.5	2.45	5	7.50	12.25	$262.50	$857.50	$1,120.00
77328	2	3.87	12	24.00	46.44	$840.00	$3,250.80	$4,090.80
77331	1	2.76	18	18.00	49.68	$630.00	$3,477.60	$4,107.60
77332	0.4	0.11	41	16.40	4.51	$574.00	$315.70	$889.70
77333	0.75	0.3	50	37.50	15.00	$1,312.50	$1,050.00	$2,362.50
77334	1.2	0.34	337	404.40	114.58	$14,154.00	$8,020.60	$22,174.60
77336	0	1.5	1375	0.00	2062.50	$0.00	$144,375.00	$144,375.00
77370	0	4	22	0.00	88.00	$0.00	$6,160.00	$6,160.00

**Table II:** Equipment and total costs associated with providing radiation oncology physics routine services.

CPT code	Volume annual	Total sal cost	Equipmnt deprec.	Direct costs	Indirect costs	Total costs
77300	600	$31,710.60	$12,672.00	$44,382.00	$17,752.80	$62,134.80
77305	20	$1,848.00	$873.60	$2,721.60	$1,088.64	$3,810.24
77310	41	$4,534.60	$2,194.32	$6,728.92	$2,691.57	$9,420.49
77315	190	$28,595.00	$14,364.00	$42,959.00	$17,183.60	$60,142.60
77321	5	$773.50	$385.20	$1,158.70	$463.48	$1,622.18
77326	5	$920.50	$375.60	$1,296.10	$518.44	$1,814.54
77327	5	$1,120.00	$474.00	$1,594.00	$637.60	$2,231.60
77328	12	$4,090.80	$1,690.56	$5,781.36	$2,312.54	$8,093.90
77331	18	$4,107.60	$1,624.32	$5,731.92	$2,292.77	$8,024.69
77332	41	$889.70	$501.84	$1,391.54	$556.62	$1,948.16
77333	50	$2,362.50	$1,260.00	$3,622.50	$1,449.00	$5,071.50
77334	337	$22,174.60	$12,455.52	$34,630.12	$13,852.05	$48,482.17
77336	1375	$144,375.00	$49,500.00	$193,875.00	$77,550.00	$271,425.00
77370	22	$6,160.00	$2,112.00	$8,272.00	$3,308.80	$11,580.80

From this analysis, it is seen that the cost associated with providing 77336, the continuing medical physics consultation, is significant, reflective of the ongoing clinical involvement of medical physicists in support of clinic activities. The cost is due to the salary of the medical physicist as well as the amount of measured time per patient the medical physicist devotes to providing this service. Other high cost services are the complex isodose curve plan, 77315, and the complex device, 77334. Together, these services account for approximately 80% of the cost of a routine procedures medical physics program. Therapeutic radiology simulation—77295 is not listed in Tables I and II, as three‐dimensional (3D) reconstruction and treatment planning is considered a special procedure, subject to additional analysis below.

Special procedures require some additional analysis. Most radiation oncology special procedures have no specific code descriptors, so existing codes must be modified or additional information attached in order to avoid payment denial. An exception to this is Three‐Dimensional Treatment Planning, which is associated with CPT code 77295. The physics and dosimetry work associated with a number of radiation oncology special procedures was measured and reported in the *Survey of Physics Resources for Radiation Oncology Special Procedures*,^5^ published by the American College of Medical Physics. A CPT code based analysis is not appropriate for special procedures, since equipment costs are greater and the hours per service are much greater and more variable than for routine services. A financial analysis of these services is listed in Table III and IV.

**Table III:** Salary costs associated with radiation oncology physics special procedures.

Special procedure	Patients per year	Dosim hours	Phys hours	Sal cost dosim	Sal cost phys	Total sal cost
Total skin electrons	4	5	14	$700.00	$3,920.00	$4,620.00
HDR brachytherapy	58	2	9	$4,060.00	$36,540.00	$40,600.00
Total body irradiation	26	3	10	$2,730.00	$18,200.00	$20,930.00
Linac stereotactic radiosurgery	27	3	12	$2,835.00	$22,680.00	$25,515.00
Intraoperative radiotherapy	19	1	8	$665.00	$10,640.00	$11,305.00
Electron arc irradiation	4	9	22	$1,260.00	$6,160.00	$7,420.00
Stereotactic brachytherapy	5	3	19	$525.00	$6,650.00	$7,175.00
3D treatment planning	133	7	6	$32,585.00	$55,860.00	$88,445.00

Patients per year are the average number of patients treated by these special procedures in the clinics where these services are offered. In this analysis, medical physicist compensation start‐up costs are addressed by allocating the start up physicist hours over a five‐year average of the patient load. In addition, radiotherapy and physics equipment costs are lumped together and depreciated over five years. This equipment is additional to that required to perform routine procedures. As an example, the median additional cost to replace (or upgrade) a 2D treatment‐planning computer with a 3D computer is listed under equipment cost for 3D treatment planning. Salary cost is again based on $35.00 per hour for a dosimetrist, and $70.00 per hour for a medical physicist. Dosimetrist and physicist hours per patient are based on the median time needed to provide ongoing support for these services plus medical physicist commissioning time distributed over a five‐year patient load. Annual salary cost and equipment depreciation are totaled as direct costs. Indirect costs are again estimated as 40% of direct costs, however the financial officer must defend any indirect cost ratio with documentation. Total cost information, reported by example in Table IV, may be used to defend the expense of providing these radiation oncology special procedures to a community.

Special procedure costs are variable, with HDR‐brachytherapy, linac based radiosurgery, and three‐dimensional treatment planning being the most expensive services to provide. This is due to the high equipment costs associated with delivering these treatments. However, the cost per patient is mitigated by the larger volume of patients treated with these procedures and the associated revenue.

**Table IV:** Equipment and total costs associated with radiation oncology physics special procedures.

Special procedure	Total sal cost	Equip. cost	Equip. Deprec.	Direct costs	Indirect costs	Total costs
Total skin electrons	$4,620.00	$3,165.00	$633.00	$5,253.00	$2,101.20	$7,354.20
HDR brachytherapy	$40,600.00	$493,080.00	$98,616.00	$139,216.00	$55,686.40	$194,902.40
Total body irradiation	$20,930.00	$3,750.00	$750.00	$21,680.00	$8,672.00	$30,352.00
Linac stereotactic radiosurgery	$25,515.00	$290,170.00	$58,034.00	$83,549.00	$33,419.00	$116,968.60
Intraoperative radiotherapy	$11,305.00	$15,400.00	$3,080.00	$14,385.00	$5,754.00	$20,139.00
Electron arc irradiation	$7,420.00	$22,500.00	$4,500.00	$11,920.00	$4,768.00	$16,688.00
Stereotactic brachytherapy	$7,175.00	$27,500.00	$5,500.00	$12,675.00	$5,070.00	$17,745.00
3D treatment planning	$88,445.00	$380,625.00	$76,125.00	$164,570.00	$65,828.00	$230,398.00

Equipment cost reflects additional physics equipment purchased to perform the special procedure. The high equipment cost for 3D treatment planning and HDR brachytherapy reflects the cost of the treatment planning system. For this analysis, we allocated one‐half of the total cost of the HDR system to the physics cost center to reflect the approximate cost of the treatment‐planning computer. There was a wide spread in the data among the institutions measured, reflecting different institutional approaches to quality of treatment as well as experience of the physicist and dosimetrist. These data are used for illustration only, and actual institutional costs should not be compared with those in this report. Many institutions offer only one or two of these procedures, and many of the institutions offering the techniques have low utilization in terms of patients per year.

## RADIATION ONCOLOGY CODING

There are difficulties in charge coding for radiation oncology, and these arise for a number of reasons. Present standards of clinical practice require that each patient's radiation treatment be customized to fit his/her particular condition. There are significant variations in the amount of effort and resources used, and graduated levels of charges have been established to allow for more precise and equitable billing. The existence of several levels of the same procedure code adds complexity and difficulty to the coding process. It is difficult to construct a clinic specific manual with rules to anticipate all variations of service and levels of service provided for every patient. In some instances the present AMA Physicians' CPT coding descriptors have not kept up with new treatment techniques, and the “best fit” among existing codes must be used for billing purposes.^1^ This is particularly true for the facility services required for radiation oncology special procedures such as: total skin electron irradiation, total body irradiation, electron arc irradiation, intraoperative irradiation, high dose rate remote afterloading brachytherapy, stereotactic brachytherapy, stereotactic radiosurgery, and three‐dimensional treatment planning. In a few cases new codes have been provided, but in many cases it is necessary to provide code modifiers or additional documentation to avoid payment denial.

Basic to proper coding of charges and frequency is the assignment of the correct diagnosis code to each patient beginning a course of radiation treatment. These codes must be selected from the current *ICD‐9‐CM Coding Manual*,^6^ and must be precise as to the site and classification of disease. Medicare and some commercial insurance carriers use the ICD code to determine whether a service rendered the patient is medically necessary, and to justify the level of reimbursement for the service. Since under the False Claims Act an honest error in coding which results in a greater reimbursement carries the same penalty as miscoding, the mechanism an institution uses to establish the ICD code for patient claims filing must be examined carefully and reviewed with regularity.

In 1998, HCFA launched a number of major efforts, which have great significance for daily billing operations in the clinic. A new diagnosis coding system, ICD‐10‐PCS, is scheduled for release in 2000. Each procedure charged must carry a seven‐digit code, which is designed to furnish information more quickly to Medicare auditors about the patient's condition and about the type and number of resources used in delivering services. A second major HCFA effort involves expansion of documentation requirements and standardization of patient chart notations. These changes are directed toward elimination of payment for medical services unless the physician in the patient's chart has documented medical necessity for the particular service rendered.

Both civil and criminal penalties for Medicare fraud or abuse (intentional or unintentional) are included in the False Claims Act and other statutes. The federal government has taken the position that approximately 10% of all United States health care spending is based on illegal billing practices. New budget appropriations were made by Congress to support employment of additional examiners for a stepped up program of auditing hospitals and physicians. Software programs such as the Part B Correct Coding Initiative Edits program have been made available for their use in auditing hospital Medicare claims reimbursement. The following short description of the CCI Edits program is provided for insight.

Part B carriers are required to process each patient claim through a computer program entitled “Correct Coding Initiative,” which compares, or edits, each procedure on the claim form against a set of predetermined conditions for payment or denial. The program, developed by AdminisStar Federal for the Health Care Financing Administration (HCFA), is upgraded from time to time. The latest package of Correct Coding Initiative (CCI) Edits, Version 5.1, was downloaded from the HCFA Data Center to the Carriers in February 1999 and effective April 1, 1999.

The CCI program is not available to providers as HCFA feels attempts will be made to circumvent the conditions for denial of payment. Although the program as a whole is not available, certain elements become obvious because of denial explanations on the Medicare Explanation of Benefits form. The CCI Edits program follows a series of steps in automatically reviewing a claim prior to payment. Following are examples:

(1) It looks at the diagnosis code for the procedure charged, and checks the patient's sex. A diagnosis code for cancer of the prostate for a female patient automatically edits out the entire claim. Other items such as the patient's age are used to check the plausibility of certain diagnoses. The diagnosis code is paired with each procedure code; certain pairs are judged inappropriate treatment; and reimbursement is disallowed. These pairs are referred to as “edit pairs” by the CCI program.

(2) Each line item on a claim carries a date and if two procedures of the same kind are shown on the same date, they are ruled mutually exclusive. Certain other procedures similar to each other are considered mutually exclusive on the same day, and the program rejects the most expensive procedure and pays the least expensive one. Payment of certain services is permitted only once during a course of treatment, or perhaps only once a week.

(3) There are occasionally legitimate reasons for performing duplicate procedures on the same day. BID treatments and a double primary with both sites being treated daily are examples of this circumstance. In the case of a double primary there must be more than one diagnosis code shown on the claim form. In the absence of a second diagnosis code, such as treatment of multiple myeloma for which there is only one diagnosis code, but nevertheless treatment of three or four sites daily, a modifier must be affixed to the additional treatment charges to enable them both to pass the edit screen.

The CCI Edits program contains over 300 edit pairs to detect errors in charge coding. Continued incorrect coding, although done unintentionally, has been ruled Medicare abuse, and is subject to penalty. The complexity that is inherent in charge coding for radiation oncology has been increased many fold by the necessity to comply with the rules of editing. It is therefore necessary for any institution to develop an in‐house manual of coding rules for each of the procedures for which it bills. This manual should reflect knowledge of community standards respecting routine service billing policy, and a self‐consistent methodology for billing special procedures according the resources expended. Such a manual should be viewed as an ongoing project, always subject to revision with changing technology and treatment patterns. A sample billing manual section for the 773XX codes is reported in Appendix I. In the event of inadvertent error in billing, the manager is responsible for giving immediate credit either to the patient or insurance carrier involved. The manager must implement reporting authority and quality control for billing errors. Billing meetings including training sessions must be documented as purpose of the meeting, attendees, length of the meeting, and the manager responsible.

In addition, each institution should develop a comprehensive in‐house compliance program and distribute a compliance manual. Each service requested by a physician must be documented with written requests and the individual providing the service must sign the report of each service. The institution must continually review any charges denied by the local Medicare carrier to assure billing procedures are proper. Management must be prepared either to defend these charges or bring their billing practices into agreement with Medicare claim policies.

## DISCUSSION

One factor often overlooked is the number of services provided for patients, which are not billed or charged to the patient. There could be several reasons for this. If a linear accelerator is unavailable for treatment, it may be necessary to calculate or plan a patient for treatment on another machine. This work is usually not charged to the patient since the patient is not at fault if a treatment machine is unavailable. Protocol patients may require planning significantly beyond the effort supplied to patients not on protocol. Another possibility is if a patient were planned for treatment, but for some reason no treatment could be given. In these cases, the patient is not responsible in any way financially. Nevertheless, it is important for managers to provide local no‐charge billing codes or categories. The work performed represents real work and real effort on the part of personnel, and must be captured in order to defend staffing needs and other resources. It is not unusual to find 15–25 % of the total work performed by dosimetrists to be associated with procedures clearly identified, but not billed to the patient for one reason or another. Any such percentage will be individual to a specific clinic and must be documented before it may be used in a costing report. Medicare and other carriers view work performed on a patient but not billed to be medically unnecessary. It is therefore important to document properly all services performed on a patient even if no charge is issued.

There is an opportunity for medical physicists and dosimetrists to participate with the manager and financial officer in the generation of cost data. Financial officers usually are unaware that such detailed survey information is available respecting the equipment, time and hourly costs of providing medical services. The method outlined in this report can be used by the financial officer as line item information in the preparation of a cost report for Medicare, or similar carrier. Since the quality of the clinical program depends on appropriate reimbursement for services, it is in everyone's interest that this information be shared between the management team.

It is vitally important that managers and institutional financial officers have information, which will provide a detailed cost accounting for their business. Medicare and other health care carriers have embraced managed care as the essential solution to rising health care costs. Only if financial officers have valid cost data will they be able to persuade carriers that the costs for services are legitimate and necessary. Insurance carriers also face competition, and if they are unable to provide competitive and full service, they will suffer in the marketplace. Consequently, carriers usually are willing to support expensive cancer programs, but only if the costs are appropriately justified. Almost any service rendered by a hospital institution or a medical professional results in a charge, for which the patient is responsible. Billing is a professional act, and should be precise, accurate, and subject to the same quality controls as any other aspect of medical service or practice. The Health Care Financing Administration (HCFA) has taken the position that significant savings in health care expenditures could be affected by the elimination of reimbursement abuse and fraud. The Office of the Inspector General has issued voluntary provider guidelines for ensuring proper billing in the *OIG Compliance Program Guidance for Hospitals*.^7^ While the guidelines are considered voluntary, it is nevertheless the responsibility of the providers to exert effort to ensure compliance with all Medicare statutes and regulations. During an audit, the Medicare representative probably will be looking for evidence that some effort has been made to limit fraud and abuse. The presence of an IOG compliance program demonstrates an institution's strong commitment to honest and responsible provider and corporate conduct.

## CONCLUSION

Technical and professional billing associated with radiation oncology is highly complex, involving over 100 codes to bill to many varied routine and special procedures. Keeping such an operation cost‐effective and compliant involves great attention to detail. The resources involved in providing radiation oncology services are subject to critical analysis, and a means to defend costs has been exemplified in this report. In addition, managers bear the responsibility to train all personnel in correct billing procedures. Institutional policies must be reviewed continually both to assure a high level of billing performance and to avoid any hint of fraud or abuse of the reimbursement mechanism. The Office of Inspector General has issued guidelines for managers to establish a Compliance Program. It is highly desirable to have such a program in place, as it may indicate both desire and effort on the part of management to limit fraud and/or abuse within the institution.

## APPENDIX I: SAMPLE INSTITUTIONAL BILLING POLICIES

### 77295 Therapeutic radiology simulation‐aided field setting; three‐dimensional

Computer‐generated three dimensional reconstruction of tumor volume and surrounding critical normal tissue structures from direct CT scans and/or MRI data in preparation for non‐coplanar or coplanar therapy. The simulation utilizes documented three‐dimensional beam's eye view volume‐dose displays of multiple or moving beams. Documentation with three‐dimensional volume reconstruction and dose distribution is required.

### 77300 Basic dosimetry calculation

May be ordered for any patient calculation including MU calculations, Gap calculations, NSD/TDF calculations, off‐axis calculations, cord or other sensitive structure dose calculations, or heterogeneity calculations as prescribed by the physician. Each charge should be initialed both by the individual performing the calculations and the person checking the calculations. At least one of these persons should be either a medical physicist or a medical dosimetrist.

### 77305 Simple isodose plan

Indicates an isodose plan with one or two ports on one area of interest. Includes irregular field dose calculations with one central axis point and one to three off axis irregular field point dose calculations (parallel opposed calculations to a point in a patient count as one point. Exclude this charge for central axis point calculations, CT based plans, or plans involving wedges or custom blocking. See complex isodose plan). One charge is issued per treatment volume.

### 77310 Intermediate isodose plan

Indicates an isodose plan with three of four ports converging on a single area of interest. Simple blocking may be used to eliminate beam exposure from certain portions of the isodose plan. Includes irregular field dose calculations with between four and six irregular field point dose calculations. (Parallel opposed calculations to a point in a patient count as one point. Exclude this charge for central axis point calculations, CT based plans, or plans involving wedges or custom blocking. See complex isodose plan.) One charge is issued per treatment volume.

### 77315 Complex isodose plan

Five or more ports on a single point of interest, CT scans, arc rotation plans or stereotactic isodose plans result in this charge. Wedges, CT scans, compensators and custom bolus indicate complex plans. Custom blocking combined with CT based volume information may result in complex isodose plans being generated at the prescription plane. Electrons may be combined with photons resulting in a complex isodose plan, if photons are the most heavily weighted treatment modality. Irregular field dose calculations with seven or more irregular field point dose calculations (such as mini‐mantle or mantle fields) result in this charge. (Parallel opposed calculations to a point in a patient count as one point. Exclude this charge for central axis point calculations.) One charge is issued per treatment volume.

### 77321 Special teletherapy port plan

This code is used when an electrons only plan is used for primary treatment. It is also used when electrons are combined with photons with electrons being either the most heavily weighted beam or equally weighted with photons at a prescription point or other point of interest. This code may be used for photon hemi‐body/total body irradiation, or electron total skin irradiation. Isodose curves are not required to be associated with this charge. One charge per treatment volume.

### 77326 Simple brachytherapy isodose calculation

Manual afterloading brachytherapy source calculation: 1–4 sources. A ribbon with multiple seeds is considered one source. Remote afterloading brachytherapy calculation: 1–8 source positions. Single plane calculation only. Exclusions: Tandem and Ovoid brachytherapy. One charge per treatment volume.

### 77327 Intermediate brachytherapy isodose calculation

Manual afterloading brachytherapy source calculation: 5–10 sources. A ribbon with multiple seeds is considered one source. Remote afterloading brachytherapy calculation: 9–12 source positions. Any brachytherapy calculation involving multiple planes of calculation. Tandem and ovoid brachytherapy is considered intermediate or complex. One charge per treatment volume.

### 77328 Complex brachytherapy isodose calculation

Manual afterloading brachytherapy source calculations: 11 or more sources. A ribbon with multiple seeds is considered one source. Any volume or spatial source dose calculation. Remote afterloading brachytherapy source calculation: 13 or more source positions. All prostate seed plans. Prostate pre‐plan and post‐plan each generate one charge. One charge per treatment volume.

### 77331 Special dosimetry

This code involves the use of special radiation measuring and monitoring devices such as Thermoluminescent Dosimetry (TLD), diode dosimetry, film dosimetry, scintillation dosimetry, ion chamber dosimetry, or special dosimetry probes (such as intracavitary probes) as ordered by the Radiation Oncologist.

### 77332 Simple treatment device

Premade simple blocks, simple bolus, cork stent or premade head holder. All premade devices are included in this category. Generally reported no more than three times per treatment volume.

### 77333 Intermediate treatment device

Multiple premade blocks, custom bite blocks (stents) or special (custom) bolus. A single custom device, which is easily fabricated for an individual patient, is included in this category. (Multiple intermediate treatment devices for a single treatment site or volume result in a single complex treatment device charge—77334.) Adjustable‐custom breast patient positioning devices are included in this category.

### 77334 Complex treatment device

Irregular blocks, electron cutouts, special shields, compensators, wedges, multileaf collimators, independent jaws/asymmetric, and casts. All cerrobend cast blocks for custom irregular field blocking are included. Multileaf collimation is included. Parallel opposed field block sets (or multileaf collimator fields), as those cut from a single film and reversed for the second block, are to be charged once. However, if the planning target volume is two cm or greater removed from midline, two films are generated, and two charges are billed. It is possible, depending on the number of films generated, for a four‐field treatment to require two, three or four charges. All custom aquaplast immobilization, lead face shields with insert bolus, custom half‐beam blocks with wedges, alpha‐cradle, and vac‐lock devices are included.

### 77336 Continuing medical physics consultation

This code is used to describe ongoing medical physics service for every patient receiving radiation therapy. It includes documented weekly checking of the patient's chart by the medical physicist or by the medical dosimetrist to assure that the treatment administered confirms to that prescribed by the Radiation Oncologist.

### 77370 Special medical physics consultation

This code is used to describe a consultation in which the complexity of the treatment plan, a particular dosimetry problem, or unique clinical physics situation arises that requires the expertise of the qualified medical physicist. All aspects of the problem are analyzed and a summary is prepared for the Radiation Oncologist. The Radiation Oncologist initials the date of the physician request on the summary. The summary is signed and dated by the qualified medical physicist.
